# Endoscopic Saphenous harvesting with an Open CO2 System (ESOS) trial for coronary artery bypass grafting surgery: study protocol for a randomized controlled trial

**DOI:** 10.1186/1745-6215-12-243

**Published:** 2011-11-18

**Authors:** Antonio Campanella, Laura Bergamasco, Luigia Macri, Sofia Asioli, Roger Devotini, Serenella Scipioni, Silvana Barbaro, Pietro Rispoli, Mauro Rinaldi

**Affiliations:** 1 Thoracic and Cardiovascular Department, Division of Cardiac Surgery, San Giovanni Battista of Turin Hospital, University of Turin, Corso Bramante 84, 10126 Turin, Italy; 2 Physics Department, University of Turin, Corso Bramante 84, 10126 Turin, Italy; 3 Biomedical Sciences and Human Oncology Department, Division of Third Pathological Anatomy, San Giovanni Battista of Turin Hospital, University of Turin, Corso Bramante 84, 10126 Turin, Italy; 4 Sanitary Direction Department, Division of Hospital Hygiene and Management of Sanitary Technologies, San Giovanni Battista of Turin, Corso Bramante 84, 10126 Turin, Italy; 5 Thoracic and Cardiovascular Department, Division of Vascular Surgery, San Giovanni Battista of Turin Hospital, University of Turin, Corso Bramante 84, 10126 Turin, Italy

**Keywords:** coronary disease, minimally invasive surgery, saphenous vein harvest

## Abstract

**Background:**

In coronary artery bypass grafting surgery, arterial conduits are preferred because of more favourable long-term patency and outcome. Anyway *the greater saphenous vein *continues to be the most commonly used bypass conduit. *Minimally invasive endoscopic saphenous vein harvesting *is increasingly being investigated in order to reduce the morbidity associated with conventional open vein harvesting, includes postoperative leg wound complications, pain and patient satisfaction. However, to date the short and the long-term benefits of the endoscopic technique remain controversial. This study provides an interesting opportunity to address this gap in the literature.

**Methods/Design:**

**Endoscopic Saphenous harvesting with an Open CO_2 _System **trial includes two parallel vein harvesting arms in coronary artery bypass grafting surgery. It is an interventional, single centre, prospective, randomized, safety/efficacy, cost/effectiveness study, in adult patients with elective planned and first isolated coronary artery disease. A simple size of 100 patients for each arm will be required to achieve 80% statistical power, with a significant level of 0.05, for detecting most of the formulated hypotheses. A six-weeks leg wound complications rate was assumed to be 20% in the conventional arm and less of 4% in the endoscopic arm. Previously quoted studies suggest a first-year vein-graft failure rate of about 20% with an annual occlusion rate of 1% to 2% in the first six years, with practically no difference between the endoscopic and conventional approaches. Similarly, the results on event-free survival rates for the two arms have barely a 2-3% gap. Assuming a 10% drop-out rate and a 5% cross-over rate, the goal is to enrol 230 patients from a single Italian cardiac surgery centre.

**Discussion:**

The goal of this prospective randomized trial is to compare and to test improvement in wound healing, quality of life, safety/efficacy, cost-effectiveness, short and long-term outcomes and vein-graft patency after endoscopic open CO_2 _harvesting system versus conventional vein harvesting.

The expected results are of high clinical relevance and will show the safety/efficacy or non-inferiority of one treatment approach in terms of vein harvesting for coronary artery bypass grafting surgery.

**Trial registration:**

www.clinicalTrials.gov NCT01121341.

## Background

Since the introduction of saphenous vein grafting by René Favaloro in the 1968, coronary artery bypass grafting (CABG) surgery has become one of the most common surgical procedures performed [1]. Although approximately 15% to 20% of vein grafts occlude in the first year with an annual occlusion rate of 1% to 2% in the first six years and 4% to 5% from 6 to 10 years, the greater saphenous vein continues to be the most commonly used bypass conduit [2].

Minimally invasive endoscopic saphenous vein harvesting was introduced by Lumsden in 1995 [3,4]. Ever since it has been increasingly investigated in order to reduce the morbidity associated with conventional open vein harvesting, which requires long incisions, and thus postoperative leg wound complications, pain and patient dissatisfaction [5-7].

Data from the Society of Thoracic Surgery National Database (accessed at http://www.sts.org) show that in 2008 endoscopic harvesting was used in approximately 70% of CABG surgeries performed in USA, while in Europe its use is for various reasons still limited.

In 2005, the International Society for Minimally Invasive Cardiothoracic Surgery published a consensus statement on the use of endoscopic vein harvesting (EVH) versus open vein harvesting (CVH) in CABG aimed to determine which resulted in better clinical and resource outcomes [8]. The members of the consensus committee concluded that EVH was recommended to reduce leg wound related complications, decrease postoperative pain, accelerate postoperative mobility, improve patient satisfaction, reduce length of stay in the hospital, and use of outpatient wound-management resources; as to the quality of the conduit harvested, major adverse cardiac events (MACE) and angiographic patency at 6 months, EVH and CVH techniques were judged to fare equally.

As now, the evidence for short-term patient benefits in reducing infections and operative complications while improving patient mobility and satisfaction is sufficiently replete, including the conclusion that wound complication management following EVH requires significantly less resource utilization than for CVH and thus increase its cost-effectiveness [9].

The mid- and long-term outcome are instead still controversial issues. This may be partly due to the fact that the event-free survival rate over periods from 2 to 5 years is commonly taken as a surrogate marker for graft failure, since postoperative angiographic studies, the gold standard to evaluate the long-term influence of vein harvest technique on graft patency, are not widely used owing to their low practicality and high costs.

A recent pooled meta-analysis of all observational and randomized trials of short- to midterm follow-ups shows no differences in survival [10]. Allen et al. [11] quote as outcome of their 112 prospectively randomized patients a freedom from MACE at five years of 75% for EVH vs. 74% for CVH, which leads them to conclude that the use of endoscopic versus traditionally harvested saphenous vein does not influence event-free survival. A different opinion is instead voiced by Lopes et al. [12] who carry out a single retrospective post hoc analysis of the PREVENT IV trial, and, based on the event-free survival rates at three years of 80% for EVH vs. 83% for CVH, conclude that EVH may have a negative impact on graft patency. Actually, the relatively small gap between the event-free survival rates for the two approaches (1-3%) would require enormously large sample sizes to be established with a statistical power of at least 80%.

The comparison and discussion of EVH vs. CVH results often neglects to consider the impact of the use of endoscopic devices for vein harvesting which differ both for dissection technique and usage of CO_2. _Cheng et al. [13] underline that the Lopes' conclusions on the PREVENT IV trial may have been influenced by the fact that large part of the analysed EVH cases were performed with a closed CO_2 _system device (GUIDANT) which has a documented tendency to form clots within the vein, if heparin is not given before dissection (as it was actually the case at the times of that trial). They add other indications of device-related different outcomes: a long-term randomised controlled trial (RCT) carried out with a Ethicon (now Sorin) device showed in a 5-year follow-up no difference in MACE, while two others RCTs and a subset data of the PAS-Port proximal anastomosis system trial using Guidant (now Maquet) showed non-significant trends in worse vein-graft patency following EVH.

The device used and all the related technicalities with the ongoing improvements over the years may well be the divide among different evaluations. As a consequence, great care must be used when comparing results from trials carried out several years apart from each other.

These considerations formed the rationale for our Endoscopic Saphenous harvesting with an Open CO_2 _System (ESOS) Trial, whose protocol is herein illustrated.

## Methods/Design

The study site for this trial is the Department of Thoracic Surgery, Division of Cardiac Surgery of San Giovanni Battista Hospital-University of Turin, Italy. This center has a long-term well-established familiarity with minimally invasive techniques; from here stems the decision to extend to CABG the practice to endoscopic saphenous vein harvesting, in order to improve clinical outcomes and provide major benefits to patients.

To acquire a specific expertise in EVH with the device chosen, in the first half of 2010, prior to trial patient recruitment, there has been a 6-months break-in period dedicated to satisfactorily complete all aspects of the learning curve. 20 patients scheduled to have coronary artery bypass grafting judged amenable to the endoscopic approach were enrolled. This number is consistent with what reported by authors who went through the same learning curve process [14-16].

All results obtained in this period concur to state that the mastery of the vein harvesting technique has been successfully achieved [17,18]. The total procedure time and the number of incisions have decreased, while the vein harvest rate, or length-time index has consistently increased to reach the value of 1 cm/min suggested by Crouch et al. [19] as optimal for this method (95% CI: 0.89-1.04). Other important results have been low incidence of wound-related morbidities, absence of infection, no need for hospital re-entry or ambulatory medications after discharge, general satisfaction expressed by all patients for the prompt wound healing, the good mobility, the absence of pain and the presence of a very small scar, due to become soon scarcely discernible.

In order to ensure that during the trial all vein harvesting procedures with all their technicalities will be carried through uniformly, and thus avoid a further stratification, the vein harvesting is planned to be performed by a single surgeon (A.C.). Should other surgeons be enrolled, a careful evaluation of homogeneity of results from the different operators will be mandatory.

### Ethics and Trials Registration

The study is funded by a 2010 grant from the Azienda Ospedaliera-Universitaria San Giovanni Battista of Turin. The trial has been approved by the local Ethic Committee and registered at the United States National Institutes of Health Clinical Trials Registry (ClinicalTrials.gov ID ***NCT01121341***), available online at http://www.clinicaltrials.gov.

### Primary end points

We have two primary end points (see Appendix 1) with different deadlines:

• a short-term one (6 weeks after CABG surgery), regarding freedom from wound complications, defined as cellulitis, edema, ecchymosis, hematoma, cellulites, drainage, necrosis, dehiscence, debridement; leg wound infection according to ASEPSIS score;

• a long-term one (2 years after CABG surgery), regarding freedom from MACE, i.e. death, myocardial infarction, recurrent angina or congestive heart failure due to vein graft failure with assessment of vein-graft patency.

### Secondary end points

The secondary end points (see Table 1) include for both arms:

**Table 1:** Study design

Inclusion Criteria:	Exclusion Criteria:
Elective planned CABG surgery	Emergency revascularization: patients with hemodynamic instability or requiring inotropic or intra-aortic balloon support

First isolated CABG surgery	Previous cardiac surgery

Adult patients (18 years and older) and competent to give informed consent	Planned concomitant valve surgery

	Bad varicous veins

	Previous safenectomy

	History of deep vein thrombosis

	History of suffered trauma on the lower extremity

	Preoperative legs immobilization

	Previous leg wound complications

	Coexisting illness with life expectancy < five years

• identification of predictors for a possible development of a harvest-site complication: preoperative demographics such as sex, obesity, diabetes, peripheral vascular disease and operative demographics such as EVH vs. CVH approach, number of bypass grafts, time on bypass, harvest site (below or above the knee), characteristics of the bandage will be considered and analyzed;

• evaluation of differences between EVH and CVH in operative time, mobility time, number of medications, hospital length of stay, readmission for leg wound complications or need for outpatient wound management resources and generally in all resources that need to be allocated;

• Evaluation of differences between EVH and CVH in the quality of the harvested venous conduits;

• Comparison of health-related quality of life (EUROQol-5D) and leg pain score and identification of factors, in addition to treatment allocation, that are associated with variations in quality of life outcomes.

• correlation of the long-term outcome with the vein histological scores determined at harvest time.

For the EVH arm only:

• effect of the harvest site, whether below or above the knee;

• effect of the vein preparation solution;

• effect of an uncontrolled distension pressure or no touch technique on vein patency and long-term outcomes;

• assessment of systemic carbon dioxide absorption.

ESOS is meant to be an interventional, single centre, prospective randomized study with an all-comers design. Each cohort will initially be formed by 115 patients. This number has been selected to balance statistical requirements and resource limitations.

In the course of the trial all consecutive adult patients (18 years and older) with elective planned and first isolated coronary artery disease (CAD) amenable to coronary artery bypass grafting surgery will be screened to establish their eligibility to both EVH and CVH on the basis of the guidelines reported in Table 1.

Patients not amenable for both treatment options, or who are expected to have a better outcome with one of the two, will not be included in the trial; neither will be the patients who refuse to be randomized because of their personal preference for one approach over the other.

Patients deemed amenable for both harvesting modalities, after having signed the informed consent, will be assigned by means of a standard web-based computer generated randomization scheme to one of the two cohorts, either EVH or CVH, and after allocation they will be included in the intention to treat analysis. An independent clinical research assistant will be responsible for randomization and treatment assignment.

The post surgery follow-up will consist of clinic visits on the sixth week after CABG surgery and at occurrence of any adverse event. Each information leading to in-patient or out-patient treatment regarding major adverse cardiac events, vein-graft failure or occlusion or other morbidity, will be collected. The number of visits required to clear up each wound complication will be used as a surrogate to determine outpatient resource utilization for wound management.

Follow-up angiograms will be performed at 18 months to explore directly vein graft longevity. This will allow not to rely totally on the indirect, and sometimes deceiving, information supplied by the event-free survival rates.

Follow-up will stretch over a minimum of 24 months. At the end of this period there will be an assessment of the state of the art based on the results obtained with the two different approaches, whose rationale will lead to the decision to either conclude or continue the follow-up.

### Trial Population, and Statistical Analysis

To assess accurately the trial, the study investigators have followed the CONSORT 2010 checklist with methodological rigour [20].

**Figure 1:**
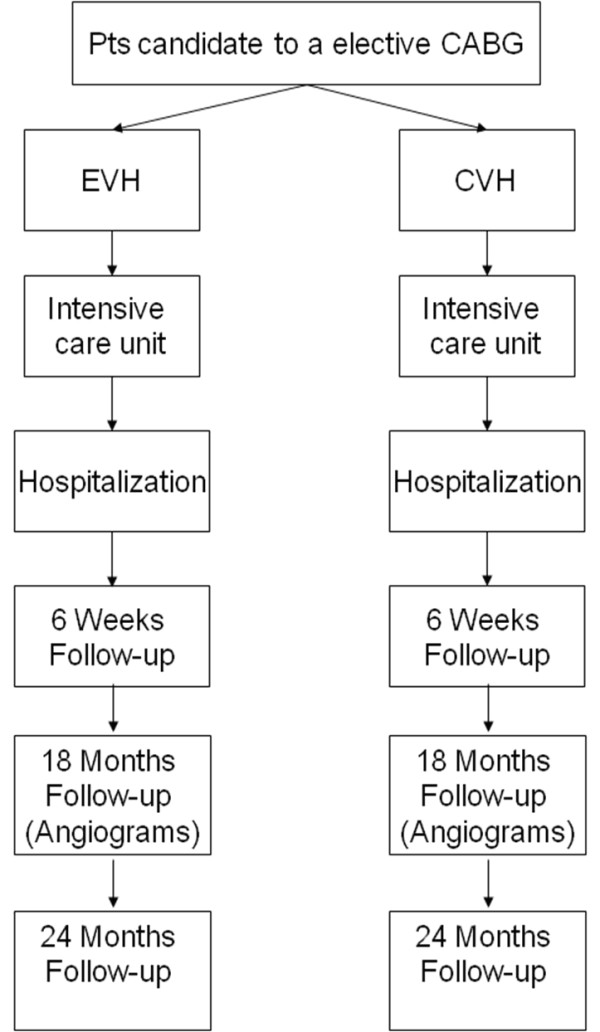
**Flowchart of the study design**. This figure illustrates the study design.

Study recruitment has initiated in August 2010. Figure 1 illustrates the planned study flowchart, and Table 1 reports its details.

All data relative to enrolled patients will be entered in a database containing preoperative demographics and risk factors (age, sex, BMI, morbidities, coronary artery lesions severity by SYNTAX score and surgical risk profile by EUROscore, etc), intra-operative variables (site of harvesting, number of incisions, lengths, times, etc), vein specific variables, pre- and post- harvesting respiratory and hemodynamic parameters, histological status of the harvested vein, as examined and graded by a pathologist. Wound healing and variables in the ASEPSIS score and other wound-related morbidities, wound complications, outcome and correlated events of the follow-up are to be evaluated by an independent clinical committee every 6 weeks in the first year, and then at 18 and 24 months. The total number of data characterizing each patient will be approximately 180, a complete scenario of his risk factors, surgical and post surgery history, short-, mid- and long-term outcome. The two cohorts will hence be described by two 115 × 180 arrays, with the intention to bring to light differences and similarities in behaviors and trends by means of a thorough statistical analysis.

The continuous data will be reported as average ± standard deviation and will be analyzed by standard ANOVA tests. Discrete or dichotomous values will be reported as percentages, risk ratios (and their 95% confidence intervals), and will be stratified in *mxn *contingency tables, analyzed for significant differences by chi-square tests (with Yates'correction in the case of 2 × 2 tables) and Fisher or mid-P tests.

The freedom from late wound complications and in general all event-free survival rates will be obtained according to the Kaplan-Meyer method, with the statistical differences among them estimated by the Mantel-Cox log-rank test. As usual, statistical significance will be determined at a *p *value (probability of null hypothesis: the observed differences are imputable to chance) ≤ 0.05, corresponding to the false positive probability (α or type I error). In case of a *p *value > 0.05, the next step will be the estimate of the test power *II *and the related false negative probability (β or type II error = 1- *II*).

A few considerations on the selected sample size are opportune. Assuming the values conventionally accepted for the drop-out and cross-over rates of ≤ 10% and ≤ 5% of the patients, follow-up will see two cohorts of approximately 100 patients for each. This means that data computed as percentage within each cohort should be correctly reported with an uncertainty of ± 5%.

It is well known that the power of the statistical test used to carry out comparisons between two sets of data depends both on the sample sizes and the entity of the difference between data observed in the two groups. A tiny difference has difficulty to be evidenced as statistical significant even with a very large sample size, while a substantial difference can be easily assessed as significant even with a small sample size.

In a trial in which the analysis wants to take into account several outcomes, the presence of a wide range of observable differences makes very difficult a "valid-for-all" estimate of the sample size necessary to achieve the desired test power (usually 80%) or inversely on the test power predicted on the base of the actual sample size.

In our trial, as far as the short-term outcome is concerned, previous studies [8,9] suggest a six-week leg wound complication rate of about 20% in the CVH arm and less than 4% in the EVH arm. With this risk/prevalence difference of 16%, and the predicted sample size of 100, the power based on normal approximation is about 94% (91% if we apply the continuity correction), and thus very good: a power of about 80% would be reached already with a sample size around 80.

The situation changes dramatically when we consider the mid- and long term outcomes. Previously quoted studies [2] suggest a first-year vein-graft failure rate of about 20% with an annual occlusion rate of 1% to 2% in the first six years, with practically no difference between the EVH and CVH approaches. On the basis of previous comparative trials of EVH versus OVH, none of the clinically relevant cardiovascular outcomes differed significantly for EVH compared with OVH (Allen et al. [11] reported survival free of MACE at up to 5 years as 75% vs. 74%, p = 0.85). A 2-3% of non-inferiority margin is therefore planned in this protocol study. The 80% test power goal, with such small risk prevalence difference requires sample sizes of at lest 3000 for each cohort, values well beyond any practical implementation in a single medical centre, at least in a lifetime.

On the basis of these considerations, the initial recruitment number of 115 patients per cohort, with end-point number of about 100 patients, sustainable from the point of view of human and financial resources, looks fully acceptable.

### Procedural techniques

The leg wound protocol established for the trial calls for all patients, irrespective of the cohort to which they have been allocated, to receive 1 g cefazolin and 1 g vancomycin hydrochloride (Vancocin) preoperatively. They will also receive a povidoneiodine (Betadine) scrub and preparatory solutions before skin incision.

Antibiotic therapy will be continued for an additional 48 hours post-operatively. All wounds will be closed at the time of heparin reversal and the administration of protamine.

### Conventional harvesting

Conventional vein harvesting will be performed through longitudinal incisions over the course of the saphenous vein (SV), starting at the medial malleolus. The length of the incision will depend on the amount of vein required (at least a minimum of 18-20 cm per each vein segment). The incisions will be closed in layers, with absorbable subcutaneous and subcuticular sutures. Elastic bandage wrapping or support stockings will be used in all patients, until up to at least 72 hours after harvesting. 

### Endoscopic vein harvesting

A 2,5 cm longitudinal incision will be made in the thigh above the knee (two finger-breadths posterior to the medial edge of the tibia). After initial identification of the saphenous vein, a plane of dissection along the anterior surface of the vein will be performed using as endoscopic dissector the Sorin ClearGlide, an open CO2 EVH system. This device is not dependent on gas insufflation to maintain tissue separation and/or the tunnel. The insufflator of the endoscopic-tower is capable to regulate the gas flow and maintain a zero gradient pressure in the cavity. A low flow of CO_2 _is required (4 l/min) to flush the working channel and optimize the vein exposure.

Vein harvesting will be initially directed proximally until 2 cm below and lateral to the pubic tubercle, where the SV enters the cribriform fascia (*fascia lata*) to join the femoral vein. Then, depending on the amount of vein required, distal (below the knee) harvesting may be extended until the medial malleolus. Branches will be divided using an electrothermal bipolar tissue-sealing cautery (Ligasure™ 5 mm Laparoscopic Instrument Valleylab). SV will be divided distally to initial incision using endoclips (Endosurgery) and endoforceps (SorinGroup), and a small stab incision will be made to pull the vein.

The incision will be closed with absorbable subcutaneous and subcuticular sutures, starting after reversal of the heparin infusion. Elastic bandage wrapping or support stockings will be used in all patients, up to at least 72 hours after harvesting.

After harvesting, the vein will be cannulated and prepared with papaverine and heparinised blood, and branches will be ligated using titanium clips or/and 3-0 silk ties.

### Histology

A first check on the validity of the endoscopic device (given for granted the surgeon's ability) may be furnished by the histological evaluation of specimens collected during the harvest, with the assessment of the level of disruption in the various layers of the venous wall. Absence, or minimal presence of disruption should constitute a good start for a satisfactory vein outcome.

The uniformity, continuity, and integrity of vein structures will be assessed by histological studies conducted in a blind fashion by two of the authors (L.M. and S.A.), who will evaluate the vein histological structures according to the so-called "Griffith score" [21] which estimates the percent disruption of each histological structure (see Table 2).

**Table 2:** Histology Grading

The histological structures to be evaluated are the:	**The numeric grading system estimates the percent disruption of each histological structure and will be scored according to the following scale **[2]:	
*endothelial layer*	0	Intact or not disruption

*elastic lamina*	1	< 10%

*medial smooth muscle and connective tissue*	2	10-25%

*adventitial connective tissue*.	3	25-50%

	4	> 50%

At this purpose it is important to point out that the results obtained on the specimens collected during the learning curve are very encouraging: the average overall score is 0.3 ± 0.6 over a total of 4.0, with a flat "zero" (intact structure) for the medial connective tissues, medial smooth muscles and adventitial connective tissues.

Another open issue is whether the vein preparation procedure might influence SV histology. As now, there is no standard vein-preparation solution used in clinical practice [22,23]. The authors will investigate whether the preparation solution and a controlled distension pressure or no touch technique might prevent histological disruption and influence vein patency and long-term outcomes. (Appendix 1)

## Discussion

Nowadays, a research to evidence benefits, harms and resource implications of EVH vs. OVH must not only meet the requirements of being prospective, randomized and including a great number of baseline, inter-operative, post-operative and follow-up co-founding variables, but needs also to exploit the latest improvements in the available technologies of vein harvesting.

The Endoscopic Saphenous harvesting with an Open CO_2 _System (ESOS) Trial, was configured after these guidelines.

Its primary goal is to compare and to quantify improvements in short and mid-term outcomes after endoscopic open CO_2 _harvesting (EVH) versus conventional open vein harvesting (CVH): safety/efficacy, leg wound infections and morbidity, patient satisfaction, short and long-term outcome, with histological and angiographic studies will be matter of thorough investigation.

The results on wound healing, mobility, patient satisfaction, health-related quality of life, safety/efficacy, cost-effectiveness are expected to be available shortly after the beginning of the trial. The mid-term outcomes and vein-graft patency will be estimated at the end of the 2-year follow-up, when a possible extension of the period of observation will also be considered.

A secondary goal of ESOS is to provide guidance to physicians on optimal CABG strategies for patients with different risk levels. A more widely spread and accurate knowledge on this issue may also help patients in their choices in a difficult predicament of their life when they have to take fundamental decisions for their health.

When compared to CVH procedure, EVH is expected to confer a 16% reduction in leg wound infections. If the use of new improved instrumentation will also improve the long-term event-free survival rate, as we hope, endoscopic saphenous vein harvesting should then be definitely recommended to patients with CAD, also for the high level of satisfaction usually reported by patients undergoing this minimally invasive procedure.

This study, besides providing new information aimed to improve the quality of care for CAD patients, will also assess the cost/effectiveness of this approach as far as in-patient and out-patient care is concerned.

### Trial status

Currently including patients

## List of abbreviations used

ESOS: Endoscopic saphenous harvesting with an open CO_2 _system; CABG: Coronary artery bypass grafting; CAD: Coronary artery disease; PCI: Percutaneous coronary intervention; EVH: Endoscopic vein with open CO_2 _harvesting; CVH: Conventional vein harvesting; SV: Saphenous vein; MACE: Major adverse cardiovascular events: death; myocardial infarction; revascularization (PCI or CABG) for ischemia or angina recurrence; GF: vein-graft failure at least 75% of stenosis at angiographic study; GO: vein-graft occlusion

## Competing interests

Under present collaboration agreements, Sorin Group Italy S.r.L. has furnished complimentary kits for the learning curve.

## Authors' contributions

AC made substantial contributions to the conception, design, drafting of this study, and he is the vein harvester surgeon. MR is the principal investigator and Chief of Division of Cardiac Surgery. RD is involved in active recruitment of patients. MR, AC, LB and PR were involved in critically revising the manuscript. SS and SB obtained funding for the study. LM and SA will participate in the histological studies. LB will be responsible for the statistical analysis. All authors read and approved the final manuscript.
